# Redox Challenge in a Cultured Temperate Marine Species During Low Temperature and Temperature Recovery

**DOI:** 10.3389/fphys.2018.00923

**Published:** 2018-07-17

**Authors:** Sergio Sánchez-Nuño, Ignasi Sanahuja, Laura Fernández-Alacid, Borja Ordóñez-Grande, Ramon Fontanillas, Jaume Fernández-Borràs, Josefina Blasco, Teresa Carbonell, Antoni Ibarz

**Affiliations:** ^1^Departament de Biologia Cel⋅Lular, Fisiologia i Immunologia, Facultat de Biologia, Universitat de Barcelona, Barcelona, Spain; ^2^Skretting Aquaculture Research Centre, Stavanger, Norway

**Keywords:** redox, dietary lipids, gilthead sea bream, glutathione redox cycle, fish culture, temperature fluctuation

## Abstract

Aquaculture is a growing industry that is increasingly providing a sizable proportion of fishery products for human consumption. Dietary energy and temperature fluctuations affect fish health and may even trigger mortality, causing great losses in fish production during winter. To better understand this unproductive winter period in aquaculture, the redox status in a cultured marine species, the gilthead sea bream, was analyzed for the first time by inducing controlled temperature fluctuations and reducing dietary lipid content. Two groups of fish (by triplicate), differing in their dietary lipid content (18% vs. 14%), were subjected to 30 days at 22°C (Pre-Cold), 50 days at 14°C (Cold) and then 35 days at 22°C (Recovery). Plasma and liver redox metabolites (oxidized lipid, oxidized protein and thiol groups), liver glutathione forms (total, oxidized and reduced) and liver antioxidant enzyme activities were measured. Reducing dietary lipid content did not affect gilthead sea bream growth, glutathione levels or enzyme activities, but did reduce the amount of oxidized lipids. A sustained low temperature of 14°C showed a lack of adaptation of antioxidant enzyme activities, mainly catalase and glutathione reductase, which subsequently affected the glutathione redox cycle and caused an acute reduction in total hepatic glutathione levels, irrespective of diet. Antioxidant enzyme activities were gradually restored to their pre-cold levels, but the glutathione redox cycle was not restored to its pre-cold values during the recovery period used. Moreover, the lower lipid diet was associated with transiently increased liver oxidized protein levels. Thus, we propose that fish should be fed a low lipid diet during pre-cold and cold periods, which would reduce oxidized lipid levels without affecting fish growth, and a higher energy diet during the recovery period. Moreover, diets supplemented with antioxidants should be considered, especially during temperature recovery.

## Introduction

Global aquaculture production has increased in recent decades to meet the increased demand for seafood and is predicted to become the most reliable supply of seafood in the future. Lipids are the main source of energy in aquaculture nutrition and are included in high amounts in fish feed (Bell and Wolfang, 2010). The development of high-energy diets in aquaculture has reduced production time and increased growth rates sparing dietary protein dietary protein content, thus decreasing feed costs (Leaver et al., 2008). However, the increase in dietary lipid content is associated with increases in fish fat deposition and changes in the redox balance. In addition to fish nutrition, fluctuation in water temperature is one of the most important problems in aquaculture, with critical temperatures triggering serious thermal stress-induced problems when such variations approach either upper or lower limits that can be fatal to some species. Indeed, increased fish mortality during winter has been reported to affect a large number of fish species (Hurst, 2007).

Gilthead sea bream (*Sparus aurata*) is one of the most common marine species in the Mediterranean area and one of the most appreciated seafood species in Europe. Cultured gilthead sea bream normally experiences water temperatures ranging from 11°C in winter to 26°C, and under intensive culture, fish are unable to perform natural migration (Davis, 1988) or escape and the temperature fall becomes critical (Ibarz et al., 2010c). Below 13°C, fish activity and growth are minimal and a drastic reduction in nourishment is observed leading to growth arrest and a halt in production (Tort et al., 1998; Sarusic, 1999; Ibarz et al., 2007b; Richard et al., 2016). Although extreme cold shock is, in some cases, the direct cause of mortality, growing evidences indicate thermal stress and starvation as the major underlying physiological conditions of the mortality of over-wintering fishes (Donaldson et al., 2008). Moreover, there is no significant thermal compensation on metabolism under cold conditions on gilthead sea bream at temperatures below 13–14°C (Ibarz et al., 2010c). In this species, when fish metabolism was directly depressed by temperature a concomitant liver oxidant insult was present; lipid peroxidation (LPO) products appeared and nitric oxide production increased in a few days (Ibarz et al., 2010a). Fish diet formulation at low temperatures is still relevant and controversial concern in sea bream. High quality lipids (fish oil rich in polyunsaturated fatty acids) promote fish growth, but diets with high lipid content in the winter or pre-winter season appear to increase the incidence of pathologies associated with low temperatures (Ibarz et al., 2010c). Previous studies on this species under controlled conditions have demonstrated that diets with high lipid content alter liver metabolism, mobilizing fat from lipid reserves to the liver and causing friable and even steatotic livers due to the accumulation of unsaturated fatty acids (Sarusic, 1999; Donaldson et al., 2008). Therefore, revising the established practice of using higher lipid contents in winter diets has already been suggested, but still not implemented (Ibarz et al., 2010c).

In gilthead sea bream, food restriction and fasting increase the activities of the main redox enzymes at warm temperatures (Pascual et al., 2003), while an acute drop in temperature increases nitric oxide production and lipid peroxidation which also affect redox pathways (Ibarz et al., 2010b). In other species, plasma and blood have been used to analyze changes in the redox pathways, since peripheral blood reflects the global health status of the individual (Costa et al., 2011). However, to the best of our knowledge, no study to date has investigated changes in redox pathways in the plasma of gilthead sea bream. It was recently reported that advanced oxidation protein products (AOPPs) form from the direct oxidation of the amino acid side chains of proteins as a consequence of the damage caused by reactive oxygen species (ROS) (Bochi et al., 2014). In freshwater fish, AOPPs have been reported to act as markers of metal toxicity in the kidney and liver (Stanca et al., 2013; Hermenean et al., 2015). There are currently no data on AOPPs and their role as markers of oxidative damage in marine species.

This work aimed to study for the first time in gilthead sea bream (1) the redox status during temperature fluctuations and (2) the benefits of reducing dietary lipid content (from 18 to 14%). Since the high content of fish oil in commercial sea bream feed might affect the oxidative status, we focused on analyzing redox metabolites in the plasma and liver (oxidized lipids, oxidized proteins, and liver glutathione) and the main antioxidant enzymes in the liver (superoxide dismutase, catalase, glutathione peroxidase, and glutathione reductase). Our findings will contribute to the little knowledge currently available on the redox response to temperature fluctuations in fish and help improve the unproductive winter period in culture conditions.

## Materials and Methods

### Animal Conditions

**FIGURE 1:**
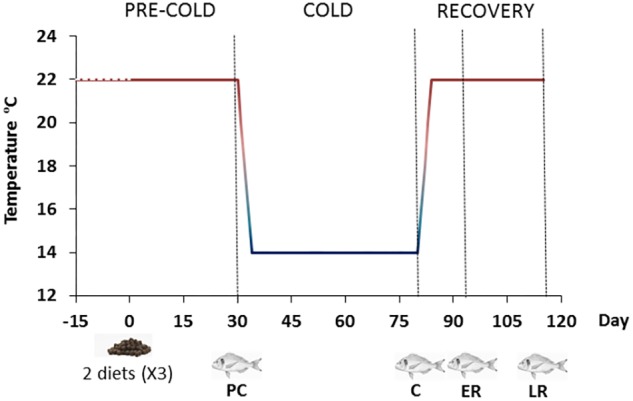
Experimental design. Experimental period (115 days) consisted of four temperature periods: pre-cold (PC) at 22°C for 30 days; cold (C), during which the temperature was cooled to 14°C over five days and maintained for a total of 50 days (including the cooling down period); early recovery (ER), during which the temperature was restored to 22°C over 5 days and measurements were taken 1 week after the start of temperature recovery; and late recovery (LR), for which measurements were taken 35 days after the start of temperature recovery. Fish symbols indicate sampling times.

Gilthead sea bream, with an average body weight of 145 ± 0.1 g, were obtained from a local fish farm and acclimated indoors at the Centre d’Aquicultura (CA-IRTA, Sant Carles de la Ràpita, Tarragona, Spain) at 22°C for 2 weeks, using a standard commercial fish feed (Skretting ARC). Following this period, they were randomly distributed into two groups per triplicate (30 fish per tank) into a water-recirculating system IRTAmar^TM^. Fifteen 500-L fiberglass tanks with solid and biological filters were used. Water temperature and oxygen concentration were monitored, while nitrite, nitrate and ammonia concentrations were maintained at initial levels throughout the experimental period. Two isoproteic diets formulated by Skretting ARC reducing their lipid content from 18 to 14% (D18 and D14, respectively) by increasing nitrogen free extract as is indicated in **Table 1**, resulting to a “crude energy” of 20.2 and 21.0 MJ/kg dry matter, respectively. Fish were fed to satiation twice a day, 7 days a week for 115 days in total. Feed was automatically delivered during 30-min sessions at 08:00 and 16:00. Satiation was ensured throughout the experimental period by calculating the estimated daily feed intake and providing a ration that was 20% above this amount. The experimental period (115 days) consisted of four periods (**Figure 1**): pre-cold (PC), during which the fish were maintained at 22°C for 30 days; cold (C), during which the temperature was cooled to 14°C over 5 days and maintained at this temperature for a total of 50 days (including the cooling down period); early recovery (ER), during which the temperature was restored to 22°C over 5 days and measurements were taken 7 days after the start of the temperature recovery period; and late recovery (LR), for which measurements were taken 35 days after the start of temperature recovery.

**Table 1:** Diets formulation and chemical composition.

Components (%)	D18	D14
Wheat	21.4	26.4
Wheat gluten	18.8	17.9
Soya concentrate	20.0	20.0
Fish meal	25.0	25.0
Fish oil	13.3	9.2
Premix vitamin and minerals	1.5	1.5
Dry matter (g/100 g)	92.4	91.8
**Nutrients (g/100 g DM)**		
Crude protein	47.0	47.0
Crude fat	18.0	14.0
NFE (nitrogen free extract)	26.0	30.0
Crude energy (MJ/kg DM)	21.0	20.2

*Information provided by Skretting ARC.*

### Fish Sampling

At the end of each temperature period, fish were fasted overnight before sampling and nine fish (3 per replicate tank and diet) were captured at random and anaesthetized with 2-phenoxyethanol (100 ppm) diluted in seawater. Body weight and length were measured, blood samples were taken from the caudal vessels using EDTA-Li as the anticoagulant, and the fish were sacrificed by severing the spinal cord. Plasma was obtained by centrifuging the blood at 13,000 ×*g* at 4°C for 5 min and samples were kept at -80°C until analysis. The weights of the livers were recorded before the samples were immediately frozen in liquid nitrogen and kept at -80°C until analysis. The study complied with the guidelines of the European Union Council (86/609/EU), the Spanish Government (RD 1201/2005) and the University of Barcelona (Spain) regarding the use of laboratory animals.

### Redox Metabolites

Lipid peroxidation was analyzed using the TBARS assay and quantified by determining the concentration of the end reactive product malondialdehyde (MDA), according to Garnacho-Castaño et al. (2016). Briefly, liver was homogenized in 10% (w/v) RIPA solution [50 M Tris (pH 7.4), 1% Triton X-100, 150 mM NaCl, 5 M NaF, 0.1% sodium dodecyl sulfate, and 1% sodium deoxycholate] containing a commercial protease inhibitor cocktail. Suspensions were centrifuged at 2,000 ×*g* for 5 min, with the resulting pellet discarded. Plasma samples for TBARS assays were pre-diluted in PBS (10% w/v). The formation of the MDA-TBA adduct was fluorometrically measured at an excitation wavelength of 515 nm and an emission wavelength of 550 nm. The calibration curve was determined with tetraethoxypropane (Slikker et al., 2001). Values are expressed as MDA in nanomoles per gram of total lipids in the plasma or per mg of fresh liver weight.

AOPPs in liver homogenates were assayed using a modified version of Witko-Sarsat et al. (2003). For sample preparation, the tissue was homogenized in PBS (10% w/v) and centrifuged at 5,000 ×*g* at 4°C for 10 min. Before testing, the liver and plasma samples were pre-diluted in PBS until a maximum concentration of 25 mg of protein per mL was obtained. AOPP formation was spectrophotometrically measured at 340 nm, involving a standard calibration curve using 100 μL of chloramine-T solution (0–100 μmol/L). AOPP concentration was expressed as chloramine-T nanomoles per mg of protein in the plasma or per mg of fresh liver weight.

Concentrations of plasma and liver total protein sulfhydryl groups or thiol groups (-SH) were determined with an adaptation of a protocol developed by Sedlak and Lindsay (1968). Samples were diluted in cold 0.25 M Tris-Base (pH 7.4) and centrifuged at 5,000 ×*g* for 10 min at 4°C. Total thiol groups were quantified from the supernatant aliquot after the addition of 150 μl of 1 mM Tris-EDTA, 10 μl of 5,5′-dithiobis(2-nitrobenzoic acid) (DTNB) and 800 μl of methanol. The mixture was homogenized and incubated at room temperature for 15 min. The endpoint reading was at 414 nm and total thiols were expressed as nanomoles per dL of plasma or nanomoles per mg of fresh liver weight.

Hepatic levels of reduced glutathione (GSH) and oxidized glutathione (GSSG) were evaluated using the procedure described by Hissin and Hilf (1976) and modified by Alva et al. (2013). Liver samples were homogenized in cold buffer containing 5 mM phosphate–EDTA buffer (pH 8.0) and 25% HPO_3_ (3.75:1). The homogenates were ultracentrifuged at 100,000 ×*g* at 4°C for 30 min and the resulting supernatants were used to determine GSH and GSSG concentrations, using the fluorescent probe o-phthalaldehyde (OPA). GSSG was previously incubated with N-ethylmaleimide to avoid interference with GSH complexes. After 15 min of incubation, fluorescence was determined at an emission wavelength of 420 nm (excitation at 350 nm) and data are shown as GSH nanomoles or GSSG nanomoles per g of fresh weight and as total glutathione (TGSH = GSH + GSSG) and glutathione power (GSH/GSSG ratio).

The total amount of proteins in the liver and plasma was determined using the (Bradford and Williams, 1976) protein assay adapted for a 96-well plate, with the Bradford reagent obtained from Bio-Rad (CA, United States) and using albumin to derive the standard curve.

### Redox Enzyme Activities

Liver samples for enzyme analyses were pulverized in liquid nitrogen and homogenized with a 1:9 (w/v) cold buffer (100 mM Tris–HCl, 0.1 mM EDTA and 0.1% Triton X-100 (v:v), pH 7.8). Homogenates were centrifuged at 25,000 ×*g* at 4°C for 30 min. After centrifugation, supernatants containing the protein extract were aliquoted and kept at -80°C until analysis.

Superoxide dismutase (SOD, EC 1.15.1.1) hepatic activity was determined using the enzymatic method of McCord and Fridovich (1969) and adapted for fish (Furné et al., 2009). A diluted aliquot was mixed with 50 mM potassium phosphate buffer (pH 7.8) containing 0.1 mM EDTA, 1 mM cytochrome C, 1 mM xanthine, 0.5 IU/ml xanthine oxidase and sodium hydrosulphite. Enzyme activity was determined by increasing optical density at 550 nm in 180-s periods. Arbitrary units were defined as one activity unit corresponding to the amount of enzyme required to produce a 50% inhibition of the ferricytochrome-C reduction rate.

Catalase (CAT, EC 1.11.1.6) hepatic activity was analyzed by following (Aebi, 1984) with minor modifications, measuring the decrease in the optical density of hydrogen peroxide (H_2_O_2_) at 240 nm. The medium used for reading CAT activity was 50 mM potassium phosphate buffer, pH 7.0, and a fresh solution of 10.6 mM H_2_O_2_.

Glutathione peroxidase (GPX, EC 1.11.1.9) activity was assayed by measuring the oxidation of NADPH at 340 nm (Bell et al., 1985). Measurements were performed in 50 mM potassium phosphate buffer (pH 7.2) containing 1 mM EDTA, 2 mM sodium azide, 0.5–1 U/ml glutathione reductase, 2 mM reduced glutathione and 0.1 mM NADPH.

Glutathione reductase (GR, EC 1.8.1.7) activity was measured by analyzing NADPH oxidation at 340 nm (Carlberg and Mannervik, 1985). Assays were performed in 0.1 M potassium phosphate buffer (pH 7.5) containing 1 mM EDTA, 0.63 mM NADPH and 3.25 mM oxidized glutathione.

All enzymatic analyses were performed in 96-well microplates at 25 ± 0.5°C with the Tecan M200 spectrophotometer (Tecan Trading AG, Switzerland). All reagents, substrates, coenzymes and purified enzymes were from Sigma (Unites States) and Bio-Rad Laboratories, Inc. (Unites States). Except for SOD (explained before), enzymatic activities are shown as IU (CAT) or mIU (GPX and GR) per g of fresh liver weight, where one unit is defined as the amount of enzyme required to transform 1 μmol of the substrate per minute under the assay conditions.

### Statistical Analysis

Statistical differences between the temperature periods for each diet were analyzed by one-way nested analysis of variance (ANOVA), with tank as the random factor to test for the possible tank effect. When there was no tank effect, ANOVA followed by Tukey’s or Dunnett’s *post hoc* test was performed when variances were uniform or not, respectively. To compare diets within each period, Student’s *t*-tests were performed. Differences were considered statistically significant when the *p*-values were <0.05. The Shapiro–Wilk test was first used to ensure the normal distribution of the data, while the uniformity of the variances was determined by Levene’s test. All statistical analyses were undertaken with commercial software (PASW version 20.0, SPSS Inc., Chicago, IL, United States).

## Results

### Effects of Temperature Fluctuations on Body Parameters and Plasma Metabolites

Body weight, body length, condition factor as well as hepatosomatic (HSI) and mesenteric fat (MFI) index were recorded (**Table 2**). There were no morphological differences at the end of each period between those fed the D18 diet and those on the D14 diet, showing that lowering dietary lipid content from 18 to 14% did not affect growth. Cold-induced growth arrest was observed in the animals maintained at 14°C for 50 days, with growth recovering after restoring the temperature to 22°C. The HSI increased at the end of the cold period and while the D18 diet had no effect during the recovery period, the D14 diet significantly lowered the HSI by half.

**Table 2:** Growth performance and redox-related metabolites in plasma and liver of gilthead sea bream throughout temperature fluctuation.

	DIET	Pre-cold (Day 30)	Cold (Day 80)	Early recovery (Day 87)	Late recovery (Day 115)
**Growth Parameters**					
Body weight (g)	D18	192.4 ± 9.9a	208.8 ± 4.4a	216.8 ± 5.7a	265.3 ± 11.4b
	D14	191 ± 3.7a	207.89 ± 5.4a	213.4 ± 8.7a	263.6 ± 7.5b
Body length (cm)	D18	18.8 ± 0.3a	19.6 ± 0.1b	19.4 ± 0.2b	21.3 ± 0.2c
	D14	18.8 ± 0.2a	19.77 ± 0.20b	19.63 ± 0.26b	21.08 ± 0.12c
Condition factor	D18	2.85 ± 0.04	2.78 ± 0.05	2.83 ± 0.05	2.74 ± 0.10
	D14	2.91 ± 0.06a	2.69 ± 0.06b	2.81 ± 0.04ab	2.87 ± 0.09ab
HSI	D18	1.91 ± 0.08	2.34 ± 0.14	2.15 ± 0.11*	1.79 ± 0.26
	D14	2.17 ± 0.12a	2.31 ± 0.10a	2.52 ± 0.13a	0.98 ± 0.15b*
MFI	D18	1.13 ± 0.17	0.88 ± 0.04	1.14 ± 0.12	1.78 ± 0.10
	D14	1.09 ± 0.14a	0.98 ± 0.17a	1.17 ± 0.08a	1.78 ± 0.12b
**Plasma Metabolites**					
Oxidized lipids	D18	91.18 ± 11.66a	48.76 ± 2.53b	105.8 ± 6.69a	76.61 ± 14.46ab
	D14	42.04 ± 3.25a*	20.74 ± 4.30b*	40.67 ± 2.42a*	28.33 ± 5.6ab*
Oxidized proteins	D18	10.66 ± 0.67	7.54 ± 0.33	7.92 ± 0.79	10.23 ± 0.92
	D14	10.65 ± 1.07a	8.31 ± 0.82b	7.84 ± 1.07b	8.19 ± 0.27b
Total thiols	D18	1.47 ± 0.12a	0.88 ± 0.08b	1.34 ± 0.07ab	1.37 ± 0.24ab
	D14	0.67 ± 0.18a*	1.19 ± 0.08b*	1.03 ± 0.13ab*	0.99 ± 0.1ab
**Liver Metabolites**					
Oxidized lipids	D18	18.75 ± 2.12a	18.02 ± 3.59a	4.44 ± 0.51b	5.29 ± 1.68b
	D14	11.43 ± 1.44a*	19.39 ± 3.73a	5.73 ± 0.68b	12.62 ± 3.11a*
Oxidized proteins	D18	2.10 ± 0.31a	3.50 ± 0.26b	3.05 ± 0.45ab*	2.74 ± 0.98ab
	D14	2.51 ± 0.15a	2.48 ± 1.10ab	5.62 ± 0.52b	3.25 ± 0.61ab
Total Thiols	D18	9.08 ± 2.85	13.09 ± 0.66	15.26 ± 1.54	15.11 ± 0.72
	D14	8.67 ± 2.04	14.65 ± 0.54	10.38 ± 3.16	15.84 ± 0.95

*Significant differences between thermal periods within each diet are indicated by lowercase letters (p < 0.05, One-Way ANOVA). Significant differences between diets at each thermal period are indicated by ^∗^ (Student’s t-test). Growth parameters: Condition Factor (Body weight/Body lenght 3 × 100), HSI, Hepatosomatic Index (Liver weight/Body weight); MFI, Mesenteric Fat Index (Mesenteric fat weight/Body weight); Plasma metabolites: oxidized lipids measured as nmols MDA/g total plasma lipid, oxidized proteins measured as nmols chloramine-T/mg total plasma protein, total thiols (nmols -SH/dl plasma); Liver metabolites: oxidized lipids measured as nmols MDA/mg fw), oxidized proteins measured as nmols chloramine-T/mg fw, Total thiols (nmols -SH/mg fw).*

To evaluate the plasma redox status, oxidized lipid levels (TBARS), oxidized protein levels (AOPPs) and total thiol groups were analyzed (**Table 2**). Plasma oxidized lipid levels were twofold higher in the animals fed the D18 diet at the end of the pre-cold period. By contrast, oxidized protein levels in the plasma did not differ between the diets. At the end of the cold period, plasma oxidized lipid and oxidized protein levels were reduced by half and around 25%, respectively, for both diets. Temperature recovery increased TBARS levels to pre-cold levels in 7 days (early recovery). Animals fed the D18 diet presented a higher concentration of circulating thiol groups at the end of the pre-cold period (twofold, *p* < 0.05). However, prolonged exposure to the cold produced different responses between the fish fed the different diets, with values decreasing in those on the D18 diet and increasing in those on the D14 diet, which reverted during late recovery.

### Effects of Temperature Fluctuations on Hepatic Redox Status

Redox metabolites in the liver during temperature fluctuations were also analyzed (**Table 2**). A reduction in the dietary lipid content of 4% decreased lipid peroxidation in the liver by 30% (during the pre-cold period), whereas no effects were found on protein oxidation and total thiol groups. In contrast to plasma, low temperatures did not reduce oxidized lipid levels in the liver, while D18 diet also significantly increased the amount of oxidized proteins. Gradual temperature restoration after the cold period transiently reduced oxidized lipid levels (by fourfold) for both diets, which could have been related to the presence of more oxidized lipids in the plasma, and increased (more than twofold) liver oxidized protein levels in those on the D14 diet.

**FIGURE 2:**
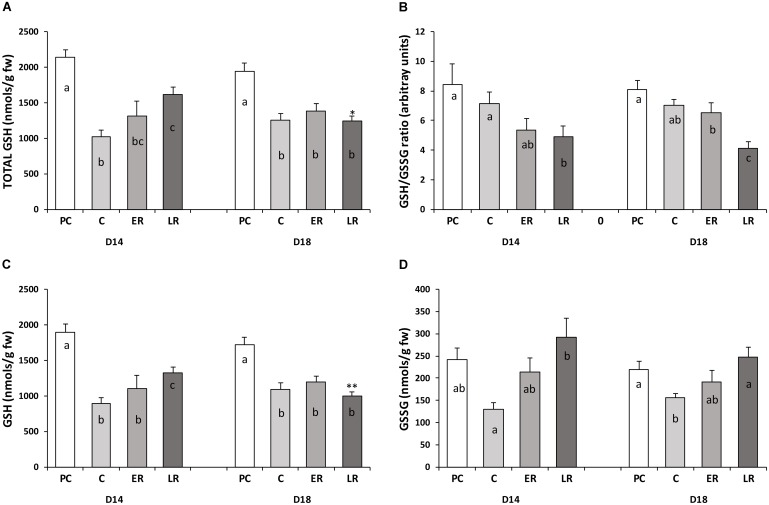
Glutathione status in the liver of gilthead sea bream throughout the study period. **(A)** Total glutathione (tGSH), **(B)** the GSH/GSSG ratio, **(C)** the reduced form of glutathione (GSH), and **(D)** the oxidized form of glutathione (GSSG). Values are mean ± standard error of the mean. PRE-COLD, data obtained from day 30 of the pre-cold period at 22°C; COLD, data obtained after 50 days at 14°C (day 80); EARLY and LATE RECOVERY, data obtained 7 and 35 days after temperature restoration to 22°C (day 87 and 115), respectively. Lowercase letters (a–c) indicate significant differences between the periods within each diet (*p* < 0.05, one-way ANOVA). Symbols (^∗^) indicate significant differences between diets at each period (^∗^*p* < 0.05 vs. D14; ^∗∗^*p* < 0.01 vs. D14; Student’s *t*-test).

At the end of the pre-cold period for both diets, total glutathione (tGSH) levels were around 2,000–2,100 nmols per gram of fresh liver weight, the reduced form (GSH) composing 90% of this amount and the oxidized form (GSSG) 10%, with a high equivalent GSH/GSSG ratio of around eight irrespective of diet. At the end of the cold period, tGSH levels decreased by half for both diets (**Figure 2A**). The GSH/GSSG ratio (**Figure 2B**) was maintained, indicating that the reduced (**Figure 2C**) and oxidized (**Figure 2D**) forms were proportionally depleted. Temperature recovery did not completely restore tGSH levels to pre-cold values, showing reductions of 25 and 40% (*p* < 0.05) for the D14 and D18 diet, respectively. Moreover, tGSH levels were significantly lower in the fish on the D18 diet than in those on the D14 diet. The GSH/GSSG ratio decreased during the late recovery period due to a gradual rise in GSSG levels because of oxidative stress that was not compensated by glutathione production.

### Effects of Diet and Temperature Fluctuations on Hepatic Enzyme Activities

**FIGURE 3:**
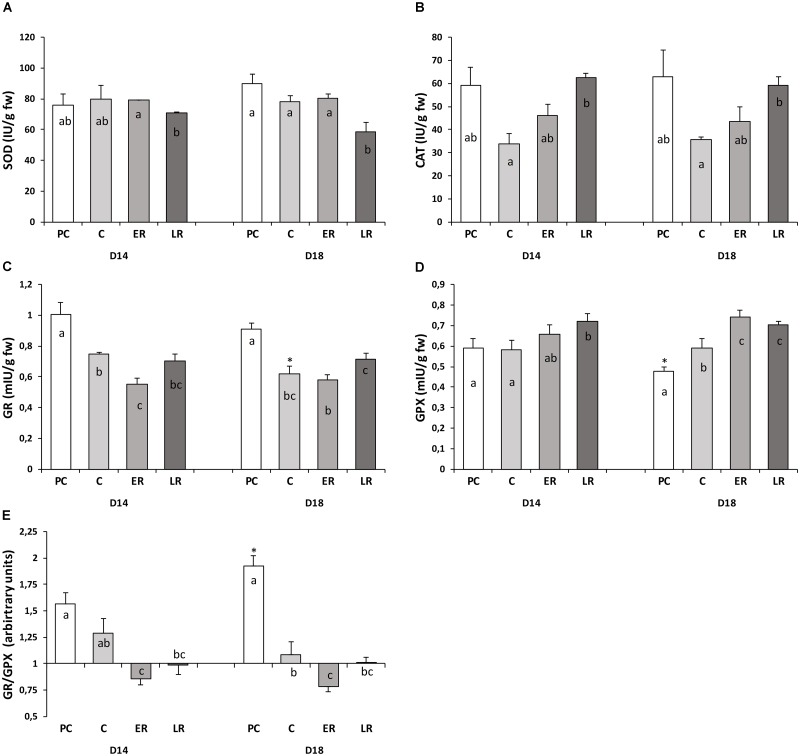
Activity of redox-associated enzymes (**A**: SOD, **B**: CAT, **C**: GR, **D**: GPX, and **E**: the GR/GPX ratio) throughout the study period. Values are mean ± standard error of the mean. PRE-COLD, data obtained from day 30 of the pre-cold period at 22°C; COLD, data obtained after 50 days at 14°C (day 80); EARLY and LATE RECOVERY, data obtained 7 and 35 days after temperature restoration to 22°C (day 87 and 115), respectively. Lowercase letters (a–c) indicate significant differences between the periods within each diet (*p* < 0.05, one-way ANOVA). Symbols (^∗^) indicate significant differences between diets at each period (^∗^*p* < 0.05 vs. D14; Student’s *t*-test).

Antioxidant enzyme activities (SOD, CAT, GR, and GPX) in response to temperature changes are shown in **Figure 3**. Liver enzyme activities were not different between the diets during the pre-cold period, except for the lower (10%) GPX activity observed in the animals fed the D18 diet that resulted in a higher GR/GPX ratio. Changes in the enzyme activities in response to the drop in temperature were due to the lack of adaptation to the cold, with no increases in the enzyme levels to compensate for the reduced metabolism caused by the low temperature. CAT and GR maximal activities were reduced by 50 and 25%, respectively, for both diets. Together with the lower tGSH amounts, these values indicate that low temperatures reduced antioxidant protection in the liver, especially in the fish fed the D18 diet, in which the GR/GPX ratio was reduced (by half), indicating an acute alteration in glutathione biosynthesis and turnover, respectively. Temperature recovery gradually restored CAT activity; however, GR activity was not fully restored to pre-cold levels. GPX activity increased during temperature recovery, its activity being 15–20% higher than during the pre-cold period. As a result, the glutathione redox cycle was not restored to its pre-cold state, the GR/GPX ratio still being lower than during the pre-cold period based on the changes observed in the GSH/GSSG ratio. The glutathione redox power, measured by both ratios (GSH/GSSG and GR/GPX), presented great variation throughout the study period, suggesting that 50 days of hypothermia at 14°C strongly affected the glutathione redox status and that 30 days of temperature recovery were not enough to mitigate the changes caused in the glutathione redox cycle.

## Discussion

A relationship has been recently reported between a winter bottleneck in fish culture and oxidative stress induced by low temperatures (Ibarz et al., 2010b,c; Richard et al., 2016). However, it is still not known whether temperature fluctuations or dietary lipid content influence the redox balance. This study analyzed, for the first time, gilthead sea bream redox status in response to a reduced dietary lipid content (from 18 to 14%) and temperature fluctuations (from 22° to 14°C and recovery from 14° to 22°C) corresponding to temperature changes that occur during a crucial period in gilthead sea bream culture (Ibarz et al., 2010c). Under our experimental conditions, fish presented similar body weight and condition factor at the end of each period regardless of diet, indicating that the animals had compensated for their lower lipid intake to maintain their energy balance. Some studies, testing the effects of increasing dietary lipid content on gilthead sea bream usually at higher temperatures (from 24° to 27°C), have reported that lipid content alone does not significantly affect fish growth (Velázquez et al., 2006; Bonaldo et al., 2010; Mongile et al., 2014). As expected, low temperatures induced “winter growth arrest,” reducing growth and ingestion, while increasing the HSI, which has also been reported by the very few studies in this field (Hurst, 2007). Some studies have highlighted the importance of understanding the recovery mechanisms in this species after a cold fasting period (Tort et al., 2004; Ibarz et al., 2007b). Our data show that a 4% decrease in dietary lipid did not modify growth parameters, although the apparent dependence of HSI on dietary energy may suggest the relevance of a higher energy supply during this period. Along these lines, our previous findings in sea bream fasted at 18°C compared to cold-induced fasting at 8°C (Ibarz et al., 2007b) demonstrated the total use of hepatic glycogen after 35 days of food deprivation at warm temperatures, whereas the lipid amount did not differ throughout the cold fasting period. In addition, 15 days of recovery from an extremely cold temperature of 8°C reverted the lipid accumulation and the HSI values to pre-cold levels. The diet used for food restoration at 18°C was formulated with 21% of lipids. Comparing those results with the present study, we could assume that well-formulated higher-energy diets would prevent changes in liver size and composition during recovery from low temperatures. Lipid peroxidation is considered a good marker of oxidative damage in fish (Tocher et al., 2002; Castro et al., 2012; Lushchak, 2016). Higher levels of dietary lipids have been associated with greater susceptibility to peroxidation-induced damage in mammals, especially for diets rich in fish oil (containing high levels of polyunsaturated fatty acids) (Sies et al., 2005). However, this has not been conclusively demonstrated in fish. In red drum (*Sciaenops ocellatus*), increasing dietary lipid content (0, 7, 14, and 21%) was shown to significantly increase oxidative stress in the liver and plasma (Craig et al., 1999), while in sole (*Solea senegalensis*), TBARS levels increased in fish fed diets with high lipid content (21 vs. 11%) (Rueda-Jasso et al., 2004). In several salmonid species, tissue oxidized lipid levels have been observed to increase following intake of high PUFA amounts (Skonberg et al., 1994; Olsen et al., 1999). Furthermore, a recent study in gilthead sea bream (Castro et al., 2016) suggested that dietary vegetable oils had a protective role against lipid peroxidation in the liver and intestine compared to fish oils. In this study, reducing dietary lipid content from 18 to 14% lowered oxidized lipid levels by half in both the plasma and liver of gilthead sea bream during the pre-cold period, decreasing the putative effects of lipid peroxidation. It is important to note that these results were obtained in animals maintained in indoor conditions. In sea cage culture, the basal levels of stress are higher because of high densities, crowding, or handling (Montero et al., 1999), which negatively impact on the growth and oxidative status of fish (Suárez et al., 2015).

Proteins are also targets of oxidation (Berlett and Stadtman, 1997) and although proteins are susceptible to greater oxidative damage than lipids, the elimination of oxidized proteins is more regulated and their accumulation is usually less evident (Gutteridge and Halliwell, 2000; Valko et al., 2007). In freshwater fish, increased amounts of AOPPs in the liver and kidney of chub (*Leuciscus cephalus*) have been linked to aquatic environmental heavy metal toxicity (Hermenean et al., 2015), whereas AOPP toxicity in gibel carp (*Carassius gibelio*) has been associated with quantum dots (nanoparticles) (Stanca et al., 2013). Our analysis of AOPPs as an oxidative stress marker is the first to be conducted in marine fish. We detected AOPP in both the plasma and liver, as has been reported for mammals (Garnacho-Castaño et al., 2016). Reducing dietary lipid content did not significantly affect AOPP levels in the plasma or liver at warm temperatures despite producing differences in the level of lipid peroxidation.

To cope with the oxidative damage resulting from metabolism, animals use non-enzymatic defenses, such as thiol groups and glutathione, and enzymes with antioxidant activity (Martínez-Álvarez et al., 2005). Higher dietary lipid content doubled the total thiol levels in the plasma, but did not boost the available glutathione levels (total glutathione) in the liver. Our observed values of around 2,000 nmols per gram of fresh liver weight are within the range reported for gilthead sea bream (Sitjà-Bobadilla et al., 2005; Pérez-Jiménez et al., 2012). Whereas total glutathione values are associated with antioxidant activity, the GSH/GSSG ratio is considered to be more indicative of the intracellular redox state, corresponding to oxidative stress in fish too (Srikanth et al., 2013). In the present study, the GSH/GSSG ratio was not affected by dietary lipid changes at 22°C, suggesting that a difference of 4% in the dietary lipid content did not elicit an imbalance in the liver glutathione forms.

Antioxidant enzyme activities in the liver of gilthead sea bream were also assessed. The relationship between dietary lipids and antioxidant enzymes has been previously demonstrated by the higher liver activities of CAT and SOD in sole fed diets containing higher lipid levels (by 10%) (Rueda-Jasso et al., 2004) and in Siberian sturgeon (*Acipenser baerii*) on diets with increased (by 6%) lipid content (Babaei et al., 2017). However, a 4% change in the dietary lipid content in our study did not alter hepatic activities of SOD, CAT, or GR, although it slightly affecting GPX hepatic activity. Our data suggest that modifying the lipid content by only 4% does not affect both non-enzymatic (mainly glutathione) and enzymatic antioxidant defenses in gilthead sea bream. Thus, the observed increase in oxidized lipid products in the D18 diet at 22°C should be taken as evidence of an incipient decoupled redox status between free-radicals and antioxidant liver enzymes, which slightly disfavors the fish fed higher amounts of dietary lipids. As diets with higher lipid content (over 18%) are currently used to improve growth in fish farms, further studies focusing on the oxidative status and glutathione synthesis of these fish are required.

### The Effect of Low Temperatures

The effects of low temperatures on gilthead sea bream have been largely studied by our group (Ibarz et al., 2010c) and are associated with reduced food intake and an overall metabolic depression proportional to the rate of temperature variation (Sala-Rabanal et al., 2003; Ibarz et al., 2007a). Exposure to a thermal shift provokes a rapid oxidant insult, marked by increased levels of liver TBARS and nitric oxide, as well as the increases of protein oxidation and proteolysis levels after acute exposure at 8°C (Ibarz et al., 2010a). Cold also provokes an associated fasting condition, and it is well known that long-term fasting causes a higher peroxidation rate in sea bream (Pascual et al., 2003). However, liver lipid mobilization and proteinaemia are both reduced at sustained low temperatures (Ibarz et al., 2010c), explaining the lower amounts of both TBARS and AOPPs in the plasma. If an early oxidative attack by ROS had occurred as a result of acute cold stress, this was reverted after 50 days at the low temperature. Arctic char subjected to a low temperature for 70 days do not display increased liver peroxidation (Olsen et al., 1999), which is consistent with our results. To our knowledge, there are no data on the effects of low temperature on liver protein oxidation. Our results show that higher dietary lipid content significantly increased AOPP levels by over 50% after 50 days at 14°C.

Increased enzymatic activities are a compensatory response for adapting to low temperatures, in what is called metabolic cold adaptation (White et al., 2012). However, animals adapting to low temperatures can increase their oxidative capacity without increasing antioxidant enzyme activities (Speers-Roesch and Ballantyne, 2005; Grim et al., 2010). We noted a lack of acclimation of antioxidant enzymes to low temperatures (14°C) in gilthead sea bream. Gilthead sea bream showed decreased oxidative and antioxidative metabolism during long-term exposure to the cold, especially CAT and GR activities, which decreased by 40 and 20%, respectively. CAT, BHMT, and GST have been reported to be downregulated in the gilthead sea bream liver proteome after an acute drop in temperature (Ibarz et al., 2010b). Moreover, we observed a significant drop in total glutathione levels (50% in both reduced and oxidized forms) at the low temperature irrespective of diet, compromising the defense against oxidative stress. Furthermore, animals on the D18 diet presented greater amounts of liver AOPP, which is of particular concern during the cold period.

### Temperature Recovery

Previous studies have highlighted the importance of understanding the recovery mechanisms in gilthead sea bream after a cold fasting period (Tort et al., 2004; Ibarz et al., 2007a). Temperature recovery coincides with a progressive restoration of the pre-cold metabolic rate (Ibarz et al., 2007a) and the activation of the energy-producing machinery that results in ROS production (Gutteridge and Halliwell, 2010). In marine fish, warming to higher temperatures (to 18°–28°C) often elicits oxidative stress due to an imbalance in the generation and removal of ROS (Feidantsis et al., 2015). Hepatic levels of oxidized lipid decreased during temperature recovery, irrespective of the diet, indicating a capacity to detox oxidized lipids accumulated during cold period. Thus, increased plasma values, mainly in early recovery, should indicate an extra-liver origin of these metabolites, which are transient and reversible later in recovery. Further studies should focus on the muscle, gut or adipose tissues as the origin of these lipid-oxidized forms in response to heat stress. There were no changes in plasma AOPP levels during the recovery period and higher liver protein oxidation in the fish on the D14 diet. In mammals, the liver is the main organ clearing AOPPs produced by the whole body and can be used to monitor oxidative stress in acute-on-chronic liver failure (Liu et al., 2012; Garnacho-Castaño et al., 2016). Thus, high liver AOPP levels during the early recovery period in fish fed the D14 diet indicate that fish on the lower energy diet suffered greater protein oxidative stress during the early stages of recovery.

Regarding liver antioxidant defenses during recovery, total glutathione levels and the GSH/GSSG ratio were not restored to pre-cold levels at the end of the recovery period because concentrations of the oxidized forms had increased. In fish, recovery from a deleterious or harmful condition, such as starvation, presents varying dynamics according to the tissues or functions studied (Nicieza and Metcalfe, 1997; Metcalfe et al., 2002; Ali et al., 2003). Glutathione synthesis from three specific amino acids (glutamate, lysine, and glycine) might be crucial for recovery in gilthead sea bream. The imbalance between the reduced and oxidized forms of glutathione (the GSH/GSSG ratio) during temperature recovery coincided with increased GPX activity, which generates the oxidized form of glutathione. During temperature recovery, SOD activity remained unchanged, CAT and GPX activities increased, and GR activity occurred at low levels corresponding to that of the cold period. Except for GR, our results agree with the data that show greater gene expression of the antioxidant enzymes at high temperatures in gilthead sea bream (Feidantsis et al., 2015). Since enzyme activity levels were provided per milligram of fresh liver weight and given that fish on the D14 diet presented a twofold lower HSI than those on the D18 diet, total hepatic antioxidant capacities were markedly reduced during the recovery period. In view of the crucial role that the glutathione redox cycle played during both the cold and recovery periods, further studies are required to explore the capacity of the liver to modify glutathione levels examining not only the glutathione oxidation-reduction cycle but also its turnover, via its specific degradation to a short-peptide and via its specific two-step enzymatic biosynthesis. The recovery period of 35 days apparently was not enough to revert redox status to the pre-cold condition. Whether this incomplete recovery is attributable to the short period or to the inadequacy of the diet during this recovery period, as seems to be the case for D14, cannot be concluded from the present study. So, paying special attention to productive conditions in this species, more experiments are necessary to better know the period of recovery from low temperatures.

## Conclusion

**FIGURE 4:**
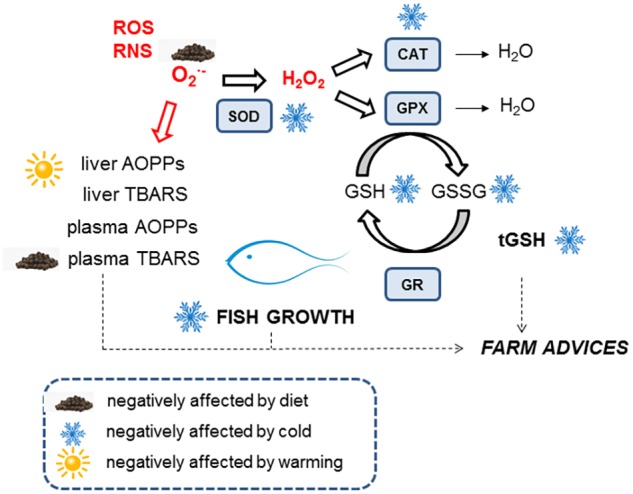
Summary of redox response throughout temperature fluctuation of gilthead sea bream. Explanation is provided in the Discussion section.

In summary, reducing dietary lipid content did not affect the growth and condition factor of gilthead sea bream grown indoors during controlled temperature fluctuations, which maintained their antioxidant defenses (glutathione and enzymes) and showed reduced oxidized lipid levels (**Figure 4**). Metabolic depression inevitably occurred at 14°C, which also affected antioxidant enzyme activities and total hepatic glutathione levels. Low energy demands at low temperatures were met by a low-fat well-balanced diet, but recovery from the cold showed a critical redox challenge due to an incomplete restoration of the glutathione redox cycle. We propose that diets with lower lipid content should be provided to cultured fish during pre-cold and cold periods to avoid excessive fat deposition and putative oxidative stress. Furthermore, protective diets containing high quality nutrients and antioxidants (including some amino acids) should be formulated.

## Author Contributions

SS-N, IS, LF-A, BO-G, TC, and AI performed the experiments. RF, JF-B, JB, TC, and AI designed the trial and diets. All authors revised the manuscript, agreed to be accountable for the content of the work, and agreed to be listed and approved the submitted version of the manuscript.

## Conflict of Interest Statement

The authors declare that the research was conducted in the absence of any commercial or financial relationships that could be construed as a potential conflict of interest.
